# Protective role of Galectin‐7 for skin barrier impairment in atopic dermatitis

**DOI:** 10.1111/cea.13672

**Published:** 2020-06-14

**Authors:** Takatsune Umayahara, Takatoshi Shimauchi, Manami Iwasaki, Jun‐ichi Sakabe, Masahiro Aoshima, Shinsuke Nakazawa, Tsuyoshi Yatagai, Hayato Yamaguchi, Pawit Phadungsaksawasdi, Kazuo Kurihara, Yoshiki Tokura

**Affiliations:** ^1^ Department of Dermatology Hamamatsu University School of Medicine Hamamatsu Japan; ^2^ Present address: Institute of Medical Biology Agency for Science, Technology and Research (A*STAR) Singapore Singapore

**Keywords:** atopic dermatitis, galectin‐7, alarmin

## Abstract

**Background:**

Atopic dermatitis (AD) patients have a barrier disorder in association with Th2 dominant skin inflammation. Galectin‐7 (Gal‐7), a soluble unglycosylated lectin, is highly expressed in the *stratum corneum* of AD patients. However, the biological significance of increased Gal‐7 expression in AD skin lesions remains unclear.

**Objective:**

We aimed to investigate the production mechanism and functional role of Gal‐7 in AD patients and IL‐4/IL‐13–stimulated epidermal keratinocytes.

**Methods:**

We assessed the Gal‐7 expression levels in skin lesions and sera from AD patients. Gal‐7 levels were also measured in monolayered normal human epidermal keratinocytes (NHEKs) and 3‐dimensional (3D)–reconstructed epidermis in the presence or absence of IL‐4/IL‐13 with or without Stat3, Stat6 or Gal‐7 gene silencing.

**Results:**

Gal‐7 was highly expressed in the *stratum corneum* or intercellular space of AD lesional epidermis as assessed by the *stratum corneum* proteome analysis and immunohistochemistry. A positive correlation was noted between serum Gal‐7 level and transepidermal water loss in patients with AD. These clinical findings were corroborated by our in vitro data, which showed that IL‐4/IL‐13 facilitated the extracellular release of endogenous Gal‐7 in both monolayered NHEKs and 3D‐reconstructed epidermis. This machinery was caused by IL‐4/IL‐13–induced cell damage and inhibited by knockdown of Stat6 but not Stat3 in NHEKs. Moreover, we performed Gal‐7 knockdown experiment on 3D‐reconstructed epidermis and the result suggested that endogenous Gal‐7 serves as a protector from IL‐4/IL‐13–induced disruption of cell‐to‐cell adhesion and/or cell‐to‐extracellular matrix adhesion.

**Conclusion and Clinical Relevance:**

Our study unveils the characteristic of Gal‐7 and its possible role as an alarmin that reflects the IL‐4/IL‐13–induced skin barrier impairment in AD.

## 1 INTRODUCTION

Atopic dermatitis (AD) is caused by an impaired barrier function of the *stratum corneum,* which is partly represented by loss‐of‐function mutation of *filaggrin* (*FLG*).^1^, ^2^ Despite the presence or absence of *FLG* gene mutation, patients with AD show an abnormal skin condition based on Th2 skewing.^3^, ^4^ Th2 cytokines, interleukin (IL)‐4 and IL‐13, directly reduce FLG expression in epidermal keratinocytes, resulting in skin barrier disruption.^5^ A skin barrier dysfunction leads to increased transepidermal water loss (TEWL) and xerosis, followed by a vicious circle of itch and scratch.^6^ Furthermore, the disrupted skin barrier allows allergic or pathogenic antigens to penetrate the epidermis more easily and stimulates epidermal keratinocytes to produce inflammatory cytokines or antimicrobial peptides, including thymic stromal lymphopoietin (TSLP), IL‐33 and β‐defensin 2.^7^, ^8^, ^9^ In particular, endogenous molecules such as IL‐33, S100, high mobility group box 1 (HMGB‐1) and β‐defensin 2 are well known as “alarmins,” which can be released from dead cells or via non‐classical secretion pathways to induce immune responses under certain inflammatory milieu.^10^

Galectin is a soluble unglycosylated lectin which its carbohydrate recognition domains bind with high affinity to β‐galactosides.^11^, ^12^ Among various types of galectin, prototypic Galectin‐7 (Gal‐7) is highly expressed in the intercellular space, cytoplasm and nucleus of epidermal keratinocytes.^13^ Importantly, Gal‐7 is involved in the homeostasis of epidermal keratinocytes, such as proliferation, differentiation, apoptosis, and cell‐to‐cell or cell‐to‐extracellular matrix (ECM) adhesion.^14^ In addition, Gal‐7 serves as a target molecule of skin resident bacteria, *Finegoldia magna*, since β‐galactosides on its cell wall have an affinity to Gal‐7.^15^

Intriguingly, Gal‐7 is highly expressed in the *stratum corneum* of AD patients and positively associated with skin barrier defects.^16^ We had previously identified and quantified 440 proteins from human *stratum corneum* by using proteome analysis of tape‐stripped skin samples.^17^ In a comparison between AD patients and healthy subjects, we have preliminarily shown the increased expression of Gal‐7 in the *stratum corneum* of patients with AD. Meanwhile, a recent study demonstrated that Gal‐7 participates in stabilization of morphological structure of epidermal keratinocytes by co‐localizing with E‐cadherin.^18^ However, the regulation and functional significance of Gal‐7 in AD skin lesions remain to be elucidated.

In this study, we first demonstrated, by using both proteome analysis and immunohistochemical (IHC) staining, that Gal‐7 was deposited in the *stratum corneum* or intercellular space of AD lesional epidermis. We also revealed the positive correlation between serum Gal‐7 levels and TEWL values in patients with AD. Our in vitro data showed that IL‐4/IL‐13 facilitated the extracellular release of Gal‐7 from normal human epidermal keratinocytes (NHEKs) and 3‐dimensional (3D)–reconstructed epidermis. It is further suggested that endogenous Gal‐7 serves as a protector from IL‐4/IL‐13–induced disruption of cell‐to‐cell adhesion and/or cell‐to‐ECM adhesion. The outcome of this study shed light on a new role of Gal‐7 as “alarmin” responding to the IL‐4/IL‐13–mediated skin barrier damage in AD.

## 2 METHODS

### 2.1 Patients’ information

For evaluation of serum Gal‐7 amounts, 20 Japanese patients with AD and 7 healthy volunteers as healthy controls (HC) were enrolled. Clinical severity of AD was assessed by SCORing AD (SCORAD) index, and barrier condition of *stratum corneum* of the cheeks was assessed by TEWL and measured by Mobile Tewameter (TM300MP, Integral). Skin biopsy samples were obtained from other 11 AD patients and 4 HC. For quantification of Gal‐7 levels in tape‐stripped *stratum corneum*, other 8 AD patients and 3 HC were also enrolled. All the patients and normal subjects were seen in September 2010 to March 2017 at Department of Dermatology, Hamamatsu University School of Medicine. AD was diagnosed according to the criteria of Hanifin and Rajka classification.^19^ All the study was conducted in accordance with the principles of the Declaration of Helsinki. Experimental protocols were approved by the medical ethical committees of Hamamatsu University School of Medicine (No. E‐14‐126‐1 and No. 17‐188). Written informed consent was obtained from the patients, and if not, the opt‐outs for the protocol were also opened to the general public by online at Hamamatsu University School of Medicine.

### 2.2 Hybrid quadrupole‐orbitrap liquid chromatography/mass spectrometry (LC/MS/MS)

All the procedure for measurement of Gal‐7 in tape‐stripped *stratum corneum* samples from both AD and normal healthy subjects by hybrid quadrupole‐orbitrap LC/MS/MS was performed as described earlier.^17^

### 2.3 Cells, cell culture and generation of 3D‐reconstructed epidermis

NHEKs were obtained from Kurabo and maintained in EpiLife medium with supplemented with S7 and antibiotic‐antimycotic (all from Gibco, Thermo Fisher Scientific). Sub‐confluent monolayered NHEKs in 6‐cm dishes were stimulated with or without indicating cytokines, IL‐4 + IL‐13 (each at 50 ng/mL), IFN‐γ (50 ng/mL) and IL‐17 + IL‐22 (each at 50 ng/mL) for 48 hours. Recombinant human IFN‐γ, IL‐17/IL‐17F and IL‐22 were purchased from R&D Systems, and recombinant human IL‐4 and IL‐13 were from Cell Signaling Technology.

A 3D‐reconstructed epidermis was generated as previously described.^20^ In brief, normal human dermal fibroblasts (NHDFs) were obtained from Kurabo and maintained in Dulbecco's minimal essential medium (DMEM) supplemented with 10% foetal calf serum (FCS) and 1% penicillin/streptomycin (PC/SM) (Wako). After polymerization of the solution on the insert at 37°C, NHDFs containing collagen solution was applied onto the insert, followed again by polymerization at 37°C. DMEM supplemented with 10% FCS, 1% PC/SM and ascorbic acid (final concentration 50 ng/mL) was added to the fibroblast‐rich gel. After 5‐day culture, the NHDFs had contracted the gel. Five days after the dermal component was prepared, 1.0 × 10^5^ NHEKs in 50 μL of EpiLife with S7 and antibiotic‐antimycotic were seeded onto the concave surface of the contracted gel. The resulting living skin equivalent (LSE) was maintained submerged in culture medium for 2 days. When the keratinocytes reached confluence, the LSE was raised to the air‐liquid interface, and cornification medium was added. The medium was changed every other day. On day 14 after airlift, 3D‐reconstructed epidermis was stimulated with or without IL‐4 + IL‐13 (50 ng/mL) for 48 hours.

### 2.4 ELISA

Patients’ sera or culture supernatants were stored at −80°C before analysis of Gal‐7 levels by RayBio^®^ Human Galectin‐7 ELISA Kit (RayBiotech) according to the manufacturer's protocols.

### 2.5 Immunohistochemical and immunofluorescence staining

The deparaffinized sections were stained with rabbit anti–Gal‐7 monoclonal antibody (mAb) (ab108623; Abcam) or anti‐E‐cadherin mAb (ab15148; Abcam) according to the manufacturer's protocols. Detection was performed by Histofine^®^ Simple Stain Max PO (MULTI) and Histofine^®^ DAB‐3S (both from Nichirei Biosciences). For quantification of intercellular Gal‐7 expression in human skin samples, the percentage of intercellular Gal‐7^+^ epidermal keratinocytes was calculated by using an image analysis software (Patholoscope; Mitani Corporation).

Monolayer NHEKs cultures were fixed in 4% paraformaldehyde. After quenching, blocking/permeabilizing and washing, cells were stained with Alexa Fluor^TM^ (AF) 488 conjugated rabbit anti–Gal‐7 mAb (ab207365; Abcam) or AF488‐conjugated rabbit IgG mAb (ab199091; Abcam). AF546‐conjugated phalloidin and AF647‐conjugated ZO‐1 were also stained (both from Thermo Fisher Scientific). After washing, cover glasses were placed on slide with Vectashield mounting medium with DAPI (Vector Laboratories). Confocal microscopy analysis was carried out using a Leica TCS SP8 (Leica Microsystems). The images were analysed using Leica Application Suite (Leica) software.

### 2.6 Cell death analysis

Sub‐confluent monolayered NHEK cultures in 6‐cm dishes were stimulated with or without IL‐4 + IL‐13 (50 ng/mL) for 48 hours. Then, cells were washed with cold PBS and stained with Annexin V Phycoerythrin (PE) Apoptosis Detection Kit I (BD Biosciences) according to the manufacturer's instructions. Dead cells, which are positive for both 7‐amino‐actinomycin D (7‐AAD) and Annexin V‐PE, were quantified by flow cytometry using a FACS Canto II (BD Biosciences).

### 2.7 RNA isolation and real‐time quantitative reverse transcriptase‐PCR (RT‐qPCR)

Total RNA was prepared from cells in culture by using PureLink RNA Mini Kit (Thermo Fisher Scientific) according to manufacturer's protocol. Next, 1 μg of total RNA was reverse‐transcribed into cDNA by using TaqMan Reverse Transcription Reagents (Thermo Fisher Scientific). Real‐time PCR was performed with a Thermal Cycler Dice Real‐Time System II (Takara Bio Inc). The relative change in the level of target gene was calculated using the 2^‐deltadeltaCT^ method. The following TaqMan probes were used: Gal‐7; Hs00170104, E‐cadherin (CDH1); Hs01023895, claudin‐1 (CLDN1); Hs00221623, desmoglein‐1 (DSG1); Hs00355084 and desmocollin‐1 (DSC1); Hs00245189 (Thermo Fisher Scientific). GAPDH; Hs99999905 (Thermo Fisher Scientific) was also amplified to normalize the variations in the cDNA levels across the different samples.

### 2.8 Western blots

Cells were lysed in RIPA buffer (Cell Signaling Technology), and the protein was separated by 10% SDS‐PAGE (both from Thermo Fisher Scientific) and transferred onto nitrocellulose blotting membranes (GE Healthcare Life Science). After blocking, staining with primary antibody and washing, the membranes were incubated with horseradish peroxidase–conjugated goat anti‐rabbit IgG antibody. The membranes were incubated with Clarity^TM^ Western ECL Substrate (Bio‐Rad), and the images were revealed with FUSION Chemiluminescence Imaging System (Vilber Lourmat). Intensities of the bands were quantified by ImageJ 1.46r. The following antibodies or kit were used: rabbit anti‐Gal‐7 polyclonal antibody (ab10482; Abcam), rabbit anti‐β‐actin mAb (#4970; Cell Signaling Technology), Stat Ab Sampler Kit and Phospho‐Stat Ab Sampler Kit (both from Cell Signaling Technology).

### 2.9 siRNA transfection

Synthetic Stat3, Stat6 siRNA oligonucleotides (SignalSilence^®^ Stat3 siRNA II, SignalSilence^®^ Stat6 siRNA II) or control siRNA (SignalSilence^®^ Control siRNA) was purchased from Cell Signaling Technology. 1 × 10^5^ cells/mL of NHEKs in 6‐cm dishes were seeded in culture medium with S7 and antibiotics, and incubated for 24 hours to allow cells to settle. After changing the medium, NHEKs were transfected with 10 nM siStat3, siStat6 or siControl using HiPerFect Transfection Reagent (Qiagen) and incubated for 24 hours. After adding another preparation of the same siRNA mix in the same way to each well, the cells were incubated for 24 hour and conducted to each experiment.

### 2.10 shRNA transfection by lentiviral particles

MISSON pLKO.1‐puro vector‐based lentiviral particles containing shRNA cassette under the U6 promoter and puromycin resistance gene were produced by Sigma‐Aldrich. As negative control, MISSON TRC2‐pLKO.5‐puro vector‐based lentiviral particles containing a non‐target shRNA (shCtr) were also used (Sigma‐Aldrich). The sequences of shRNA targeting Gal‐7 (shGal‐7) or shCtr were as follows: shGal‐7; sense 5’‐GCTCATCATCGCGTCAGACGA‐3’, antisense 5’‐TCGTCTGACGCGATGATGAGC‐3’ shCtr; sense 5’‐CAACAAGATGAAGAGCACCAA‐3’, antisense 5’‐TTGGTGCTCTTCATCTTGTTG‐3’.

On day 0, 2 × 10^5^ cells of NHEKs were seeded in 6‐cm dishes in 3 mL of EpiLife with S7 and antibiotic‐antimycotic, and the same medium was changed on day 1. On day 2, the medium was changed to 1 mL of antibiotic‐free EpiLife with S7 containing 20 μg/mL polybrene to increase infection efficiency. The lentiviral particles containing shCtr or shGal‐7 were added to the culture medium at a multiplicity of infection (MOI) of 1, and then, NHEKs were incubated for 1 hour with shake every 15 minutes. After adding 2 mL of antibiotics free EpiLife with S7, NHEKs were cultured for 24 hours. On day 3, 24 hour post‐infection, transduced NHEKs were selected in 3 mL of antibiotic‐free EpiLife containing 2.5 μg/mL puromycin. Additional 48‐h culture, NHEKs transduced with shCtr or shGal‐7 were conducted to Western blot analysis or generation of 3D‐reconstructed epidermis. In some experiments, shGal‐7–transduced 3D‐reconstructed epidermis was also pre‐incubated with a recombinant human Gal‐7 (R&D Systems) overnight at the concentration of 500 ng/mL before IL‐4/IL‐13 stimulation.

### 2.11 Statistical analysis

All statistical analyses were performed by using GraphPad Prism software version 7 (GraphPad Software). Differences between two groups were evaluated by using unpaired *t* test, paired t test or Mann‐Whitney *U* test (for non‐parametric data). When experimental groups were more than three, differences were evaluated using one‐way ANOVA with Tukey's multiple comparisons test or with Dunnett's multiple comparison test (comparisons with control group) for parametric data. For repeated data, two‐way ANOVA with Tukey's or Sidak's multiple comparison test for parametric data was used. Pearson's correlation analysis or Spearman's rank correlation (for non‐parametric data) was calculated to assess the correlation between the data. A *P*‐values < .05 was considered significant.

## 3 RESULTS

### 3.1 AD patients have increased levels of Gal‐7 in both skin lesions and sera

**Figure 1:**
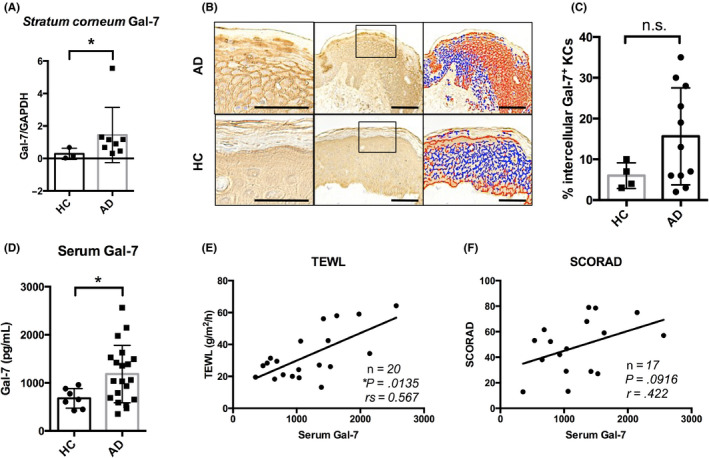
Highly expressed Galectin‐7 (Gal‐7) in both skin lesions and sera from atopic dermatitis (AD) patients.(A) Amount of Gal‐7 in tape‐stripped *stratum corneum* samples in AD and healthy control (HC) measured by liquid chromatography/mass spectrometry analysis (AD, n = 8; HC, n = 3). (B) Representative immunohistochemical staining and image analysis for Gal‐7 in skin lesions from AD and HC. The boxed areas were enlarged in the left panels. Image analyses showed the intensity of Gal‐7 expression in intercellular spaces of epidermal keratinocytes (red lines, strong positive; blue lines, middle to weak positive). Scale bars = 100 μm. (C) Percentage of intercellular Gal‐7^+^ epidermal keratinocytes in AD and HC (AD, n = 11; HC, n = 4). (D), Serum levels of Gal‐7 were measured by ELISA in AD patients and HC (AD, n = 20; HC, n = 7). (E, F) Correlation between serum Gal‐7 levels and transepidermal water loss (TEWL) (n = 20) or SCORing AD (SCORAD) (n = 17). Data were shown as means ± SD. *P < .05; n.s., not significant

We fist compared the levels of Gal‐7 expression between the AD and HC skin. By LC/MS/MS proteome analysis, we found that the amount of Gal‐7 in patients with AD was significantly higher than that in HC (Figure 1A). The IHC staining showed diffuse expression of Gal‐7 at a moderate level in the cytoplasm and nucleus of epidermal keratinocytes in normal skin (Figure 1B, lower panel). In contrast, Gal‐7 was abundantly found in the intercellular space of the epidermis and also accumulated in the *stratum corneum* of AD patient (Figure 1B, upper panel). Quantitative image analysis revealed no significant difference in the percentage of intercellular Gal‐7^+^ keratinocytes between AD and HC groups (Figure 1C **).**

We next measured serum Gal‐7 level in 20 patients with AD and 7 HC, and then examined the correlations with barrier impairment and disease severity as previously reported.^21^ In consistent with our LC/MS/MS data on the skin lesions, serum Gal‐7 level in patients with AD was significantly higher than that in HC (Figure 1D). In addition, serum Gal‐7 level positively correlated with TEWL value, a specific marker for skin barrier impairment, and tended to correlate with a clinical severity marker, SCORAD index (Figure 1E,F).

Taken together, these findings suggested that Gal‐7 was highly secreted from the epidermis and diffused into the peripheral blood, resulting in the elevation of serum Gal‐7 in patients with AD.

### 3.2 Th2 cytokines, IL‐4/IL‐13, promote Gal‐7 release from NHEKs

**Figure 2:**
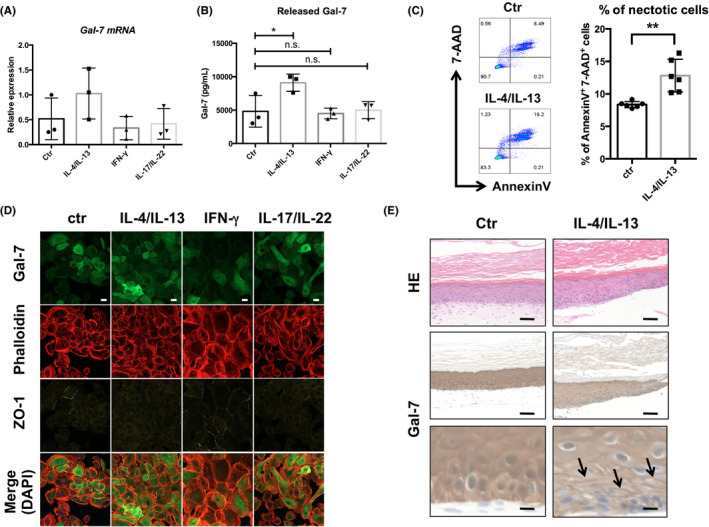
Interleukin (IL)‐4/IL‐13–mediated Galectin‐7 (Gal‐7) release from normal human epidermal keratinocytes (NHEKs). (A) NHEKs were treated with or without IL‐4/IL‐13, interferon (IFN)‐γ and IL‐17/IL‐22 for 48 h at a concentration of 50 ng/mL *Gal‐7* mRNA levels were assessed by real‐time reverse transcriptase‐PCR. (n = 3). (B) Gal‐7 protein levels in culture supernatants were measured by ELISA (n = 3). (C) IL‐4/IL‐13–induced cell death was analysed by flow cytometry (n = 6). (D) Confocal images of immunostaining for Gal‐7 (green). Nucleus, actin filaments and tight junctions were counterstained with DAPI (blue), phalloidin (red) and ZO‐1 (yellow), respectively. Scale bar = 30 μM. (E) Representative images of haematoxylin and eosin (HE) and immunohistochemical staining for Gal‐7 in 3‐dimensional (D)–reconstructed epidermis treated with or without IL‐4/IL‐13 for 48 hours at a concentration of 50 ng/mL. Scale bars = 50 μm (upper and middle) and 10 μm (bottom). Black arrows indicate intercellular deposits of Gal‐7. Data were shown as means ± SD of three independent experiments performed in one (A, B) or duplicate (C). *P < .05, **P < .01; n.s., not significant

Th2 cytokines, IL‐4 and IL‐13, are thought to play a crucial role in skin barrier dysfunction of patients with AD.^5^ However, Th1‐, Th17‐ or Th22‐derived cytokines may also be involved in the pathogenesis of AD.^22^, ^23^ Therefore, we conducted an in vitro study to address the responsible cytokines that regulate production and secretion of Gal‐7 in epidermal keratinocytes. We cultured NHEKs in the presence or absence of interferon (IFN)‐γ, IL‐4/IL‐13 or IL‐17/IL‐22 at a concentration of 50 ng/mL for 48 hours and then analysed the mRNA and protein levels of Gal‐7. Our RT‐qPCR analysis demonstrated that there was no significant difference in *Gal‐7* mRNA expression levels among each group (Figure 2A). On the other hand, the concentration of Gal‐7 in the culture supernatants was significantly higher in IL‐4/IL‐13–treated NHEKs compared with non‐treated NHEKs (Figure 2B). To clarify whether IL‐4/IL‐13 stimulation causes necrotic cell death of NHEKs, we next performed the flow cytometric analysis for apoptosis/necrosis. IL‐4/IL‐13 treatment for 48 hours significantly increased the % of Annexin V^+^ 7‐AAD^+^ necrotic dead cell at a concentration of 50 ng/mL compared with non‐treated control NHEKs (Figure 2C). Confocal image showed the stable expression of cytoplasmic Gal‐7 in non‐treated control NHEKs, and the Gal‐7 expression was not changed by any of the cytokine treatment (Figure 2D).

We further generated 3D‐reconstructed epidermis as previously described^20^ and stimulated it with IL‐4/IL‐13. In consistent with the results from AD skin lesions, the IL‐4/IL‐13–treated epidermis for 48 hours clearly showed the intercellular deposition of Gal‐7 at a concentration of 50 ng/mL compared with the control epidermis (Figure 2E). These findings indicate that IL‐4/IL‐13 primarily promotes the release of endogenous Gal‐7 from keratinocytes via necrotic cell death.

### 3.3 IL‐4/IL‐13–induced Stat6 activation regulates Gal‐7 release from NHEKs

**Figure 3:**
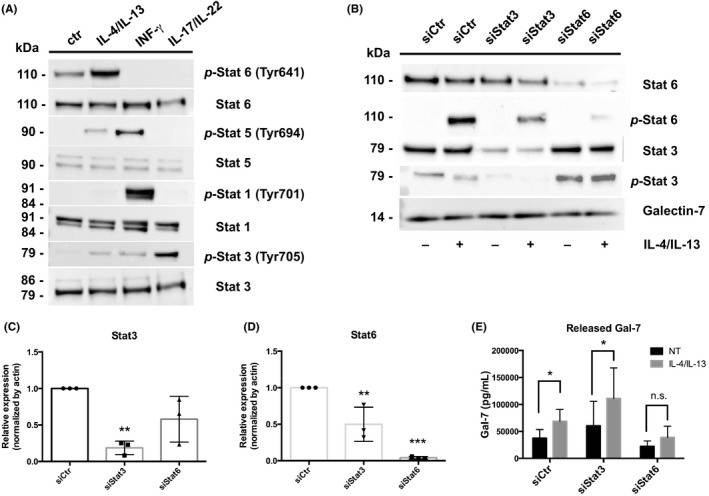
Galectin‐7 (Gal‐7) release from normal human epidermal keratinocytes (NHEKs) via interleukin (IL)‐4/IL‐13–induced signal transducers and activator of transcription (Stat)6 activation. (A) NHEKs were treated with or without IL‐4/IL‐13, interferon (IFN)‐γ and IL‐17/IL‐22 for 15 minutes at a concentration of 50 ng/mL, respectively. Western blot analyses for Stat1, phosphorylated (*p*)‐Stat1 (tyrosine [Tyr]701), Stat3, *p*‐Stat3 (Tyr705), Stat5, *p*‐Stat5 (Tyr694), Stat6 and *p*‐Stat6 (Tyr641) were performed. (B) NHEKs were transiently transfected with 10 nM small interfering (si) RNA targeting Stat3 (siStat3), Stat6 (siStat6) or non‐targeting siRNA (siControl; siCtr) for 48 hours, followed by 48‐hour stimulation with or without IL‐4/IL‐13 (50 ng/mL). Stat6, *p*‐Stat6, Stat3, *p*‐Stat3 and Gal‐7 were analysed by Western blot. (C, D) The expression values of Stat3 and Stat6 in each siRNA‐treated NHEKs were normalized to the siCtr‐treated control group (set as 1) (n = 3). (E) Gal‐7 protein levels in culture supernatants obtained from each siRNA‐treated NHEKs with or without IL‐4/IL‐13 (50 ng/mL) were measured by ELISA. Data were shown as means ± SD. *P < .05, **P < .01; ***P < .005. n.s., not significant. NT, no treatment

Both IL‐4 and IL‐13 bind to type II IL‐4 receptor, and activate Stat3 and Stat6 via janus kinase (JAK)/tyrosine kinase 2, resulting in the various biological responses in keratinocytes including spongiosis and apoptosis.^24^, ^25^ Thus, we hypothesized that IL‐4/IL‐13–induced Stat3 and/or Stat6 activation plays a crucial role in the Gal‐7 release from damaged NHEKs. First, we confirmed the IL‐4/IL‐13–induced strong Stat6 and weak Stat3 phosphorylation at a tyrosine residue in a comparison with non‐treated‐, IFN‐γ‐ or IL‐17/IL‐22–treated NHEKs (Figure 3A). We next prepared Stat3‐ or Stat6‐silenced NHEKs, and measured the released Gal‐7 in response to IL‐4/IL‐13. Western blots revealed that the transfection of siRNA for Stat3 or Stat6 was performed efficiently, and their IL‐4/IL‐13–induced phosphorylation was successfully diminished (Figure 3B‐D). We found that the Stat6 knockdown inhibited the IL‐4/IL‐13–induced Gal‐7 release from NHEKs compared with siCtr‐ or siStat3‐treated group (Figure 3E). These data suggest that Gal‐7 release from NHEKs is dependent on Stat6 phosphorylation *via* IL‐4/IL‐13.

### 3.4 Endogenous Gal‐7 serves as a protector from IL‐4/IL‐13–induced disruption of cell‐to‐cell adhesion and/or cell‐to‐ECM adhesion

**Figure 4:**
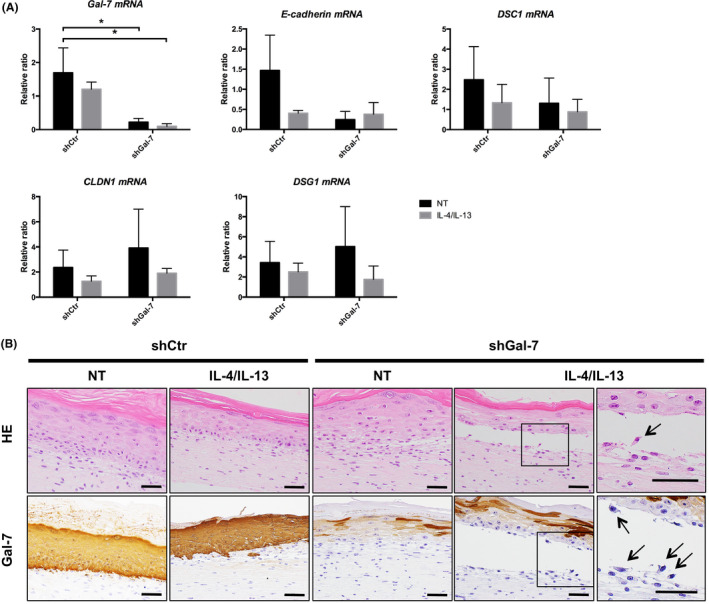
Galectin‐7 (Gal‐7) is required to avoid interleukin (IL)‐4/IL‐13–induced disruption of cell‐to‐cell and/or cell‐to‐extracellular matrix (ECM) adhesion junction of the epidermis. (A) Three‐dimensional (D)–reconstructed epidermis transduced with short hairpin control (shCtr) or shGal‐7 was treated with or without IL‐4/IL‐13 for 48 hours at a concentration of 50 ng/mL *Gal‐7*, *E‐cadherin*, *Desmocollin‐1* (*DSC1)*, *Claudin‐1* (*CLDN1)* and *Desmoglein‐1* (*DSG1)* mRNA levels were assessed by real‐time reverse transcriptase‐PCR. (B) Representative images of haematoxylin and eosin (HE) and immunohistochemical (IHC) staining for Gal‐7 in shCtr or shGal‐7–transduced 3D‐reconstructed epidermis treated with or without IL‐4/IL‐13 for 48 hours at a concentration of 50 ng/mL. The boxed areas were enlarged in the right panels. Black arrows indicate acantholysis. Scale bars = 50 μm. Data were shown as means ± SD of two or three independent experiments. *P < .05. NT, no treatment

To elucidate the functional role of IL‐4/IL‐13–induced Gal‐7 release from keratinocytes, we prepared *Gal‐7* gene silenced 3D‐reconstructed epidermis. Our RT‐qPCR showed that the *Gal‐7* mRNA was efficiently knocked down by shGal‐7 transduction; however, the other adhesion junction‐ or tight junction–associated molecules including *E‐cadherin*, *DSG1*, *DSC1* and *CLDN1* were not affected (Figure 4A).

We next investigated the morphological change in the shCtr‐ or shGal‐7–transduced 3D‐reconstructed epidermis in the presence or absence of IL‐4/IL‐13. Compared to the control epidermis, Gal‐7 knockdown epidermis had a coarse cell‐to‐cell adhesion at the basal layer or lower epidermis (Figure 4B; upper panel). Interestingly, under IL‐4/IL‐13 stimulation, shGal‐7–transduced epidermis showed a substantial spongiosis, acantholysis or even acantholytic bulla formation (Figure 4B; HE, black arrow). IHC staining confirmed that Gal‐7 expression was diminished at the basal and suprabasal layer in the shGal‐7–transduced epidermis compared with the shCtr epidermis (Figure 4B, lower panel).

We next hypothesized that extracellular Gal‐7 contributes to stabilization of IL‐4/IL‐13–induced, E‐cadherin–mediated cell‐to‐cell and/or cell‐to‐ECM adhesion. However, we could not detect significant reduction in E‐cadherin expression at the basal and suprabasal layer of the shGal‐7–transduced epidermis compared to shCtr epidermis with or without IL‐4/IL‐13 treatment (Figure S1A). Furthermore, recombinant Gal‐7 supplement had no effect on the expression levels of either Gal‐7 or E‐cadherin in the shGal‐7–transduced epidermis (Figure S1B). Thus, our results do not support for the notion that exogenous Gal‐7 stabilizes the IL‐4/Il‐13–induced, E‐cadherin–dependent disrupted keratinocyte adhesion.

## 4 DISCUSSION

In this study, we showed that Gal‐7 was released from epidermal keratinocytes and highly accumulated at the intercellular space and/or the *stratum corneum* in AD skin lesions. Consistently, a previous study showed that the amount of Gal‐7 in tape‐stripped *stratum corneum* from patients with AD was significantly higher than that from normal subjects and positively correlated with the disease severity or TEWL.^16^ We further clearly demonstrated that serum Gal‐7 level was elevated in AD patients and reflected their skin barrier impairment. As for the other clinical severity markers, we also found that the serum Gal‐7 level positively correlated with the serum levels of activation‐regulated chemokine (TARC) and lactate dehydrogenase (LDH), circulating eosinophil percentage and visual analogue scale (VAS) of pruritus (Figure S2). Thus, we indicate that the serum Gal‐7 level is a potential biomarker for AD.

NHEKs constantly express Gal‐7 in the cytoplasm and nucleus for maintaining the homeostasis and release it extracellularly to evoke biological responses to glycoproteins expressing other cell surface.^13^ Our in vitro study provides supportive evidence that Th2 cytokine IL‐4/IL‐13 may promote this machinery *via* Stat6 activation. Since we found no evidence that IL‐4/IL‐13 serves as a direct inducer of Gal‐7 transcription, we proposed that IL‐4/IL‐13–induced Stat6‐mediated cell damage might have a crucial role in the endogenous Gal‐7 release from NHEKs. As mentioned in introduction, alarmins are categorized as endogenous molecules, which are released from dead cells or via non‐classical secretion pathways, and activate the immune responses.^10^ From this point of view, it has been reported that Gal‐7 exerts an effect on T cells to proliferate, polarize towards Th1 cells and even down‐modulate their cytokine production.^26^, ^27^ Taken together, Gal‐7 could be considered as an alarmin that responds to the IL‐4/IL‐13–mediated tissue damage, as seen in Gal‐3 and Gal‐9.^28^, ^29^

IL‐4/IL‐13 can inhibit the expression of differentiation, cell adhesion or tight junction molecules of epidermal keratinocytes, such as FLG, loricrin, involucrin, keratin 1 (KRT1), KRT10, E‐cadherin, DSG1, DSC1 or CLDN1.^30^, ^31^, ^32^, ^33^, ^34^ In particular, IL‐4/IL‐13 decreases the expression of membrane E‐cadherin, and simultaneously increases the intercellular accumulation of hyaluronan (HA), which is a key pathogenic event of spongiosis.^34^ A recent study clearly demonstrated that Gal‐7 stabilizes E‐cadherin at the plasma membrane by restraining its endocytosis, and Gal‐7 silence decreases E‐cadherin–mediated intercellular adhesion.^18^ In addition, Gal‐7 is also thought to interact with ECM via β1‐integrin or matrix metalloproteinase‐9 (MMP‐9) in other cell types.^14^ Indeed, in our 3D‐reconstructed epidermis with Gal‐7 silence, the adhesion junction was disrupted at the basal layer by IL‐4/IL‐13, resulting in the formation of spongiosis and acantholysis. Our system could not provide evidence that extracellular Gal‐7 is indispensable for the stabilization of IL‐4/IL‐13–induced, E‐cadherin–mediated disrupted adhesion of keratinocytes. Nevertheless, the possibility remains that endogenous and/or exogenous Gal‐7 plays an important role in the interaction between keratinocyte and ECM to avoid adhesion disturbance under Th2 condition. Further in vivo investigation using AD mouse models might clarify whether Gal‐7 release from epidermis is a protective response from IL‐4/IL‐13–induced skin barrier impairment.

In conclusion, we showed here that IL‐4/IL‐13–induced Gal‐7 release from keratinocytes reflects the skin barrier impairment in AD patients. Elucidation of the biological and immunological role of Gal‐7 will strengthen its characteristics as alarmin. We suppose that Gal‐7 may be a potential therapeutic strategy for AD.

## CONFLICT OF INTEREST

The authors declare no competing financial interests.

## AUTHOR CONTRIBUTIONS

TU, TS, J‐I S and YT designed the study; TU, TS, MI, J‐I S, PP and KK performed experiments and analysed the data; MA, SN, TY and HY provided methodological expertise, resources and experimental assistance; TS and YT supervised the experiments; and TU, TS and YT wrote the paper.

## Supporting information

Fig S1‐S2
