# Supplementary material for: Protective role of Galectin‐7 for skin barrier impairment in atopic dermatitis
Source: Clin Exp Allergy. 2020 Jun 14;50(8):922–31. doi: 10.1111/cea.13672 (PMC7496409; doi:10.1111/cea.13672)

**SUPPLEMENTAL INFORMATIONS**

**Protective role of Galectin-7 for skin barrier impairment in atopic dermatitis**

Takatsune Umayahara, Takatoshi Shimauchi, Manami Iwasaki, Jun-ichi Sakabe, Masahiro Aoshima, Shinsuke Nakazawa, Tsuyoshi Yatagai, Hayato Yamaguchi, Pawit Phadungsaksawasdi, Kazuo Kurihara, Yoshiki Tokura

Online Data Supplement

**Supplemental Figures**

**Supplementary Figure S1.** Lack of evidence that exogenous galectin-7 (Gal-7) stabilizes the interleukin (IL)-4/IL-13-induced, E-cadherin-mediated disruption of cell adhesion of the epidermis. A, Representative images of immunohistochemical (IHC) staining for E-cadherin in short hairpin control (shCtr) or shGal-7 transduced 3-dimension (D)-reconstructed epidermis treated with or without IL-4/IL-13 for 48 h at a concentration of 50 ng/mL. B, Representative images of IHC staining for Gal-7 and E-cadherin in shCtr or shGal-7 transduced 3D-reconstructed epidermis treated with IL-4/IL-13 for 48 h at a concentration of 50 ng/mL in the presence or absence of pre-incubation with a recombinant human Gal-7 (rhGal-7; final concentration of 500 ng/mL). Scale bars = 50 μm.

**Supplementary Figure S2.** Serum levels of Galectin-7 (Gal-7) positively correlate with disease activity markers in atopic dermatitis (AD) patients. Correlation between serum Gal-7 levels and serum thymus and activation-regulated chemokine (TARC) (n=17), serum lactate dehydrogenase (LDH) (n=17), % eosinophils (n=17), and Visual Analogue Scale (VAS) of pruritus (n=20). ***p* < 0.01; ****p* < 0.005.


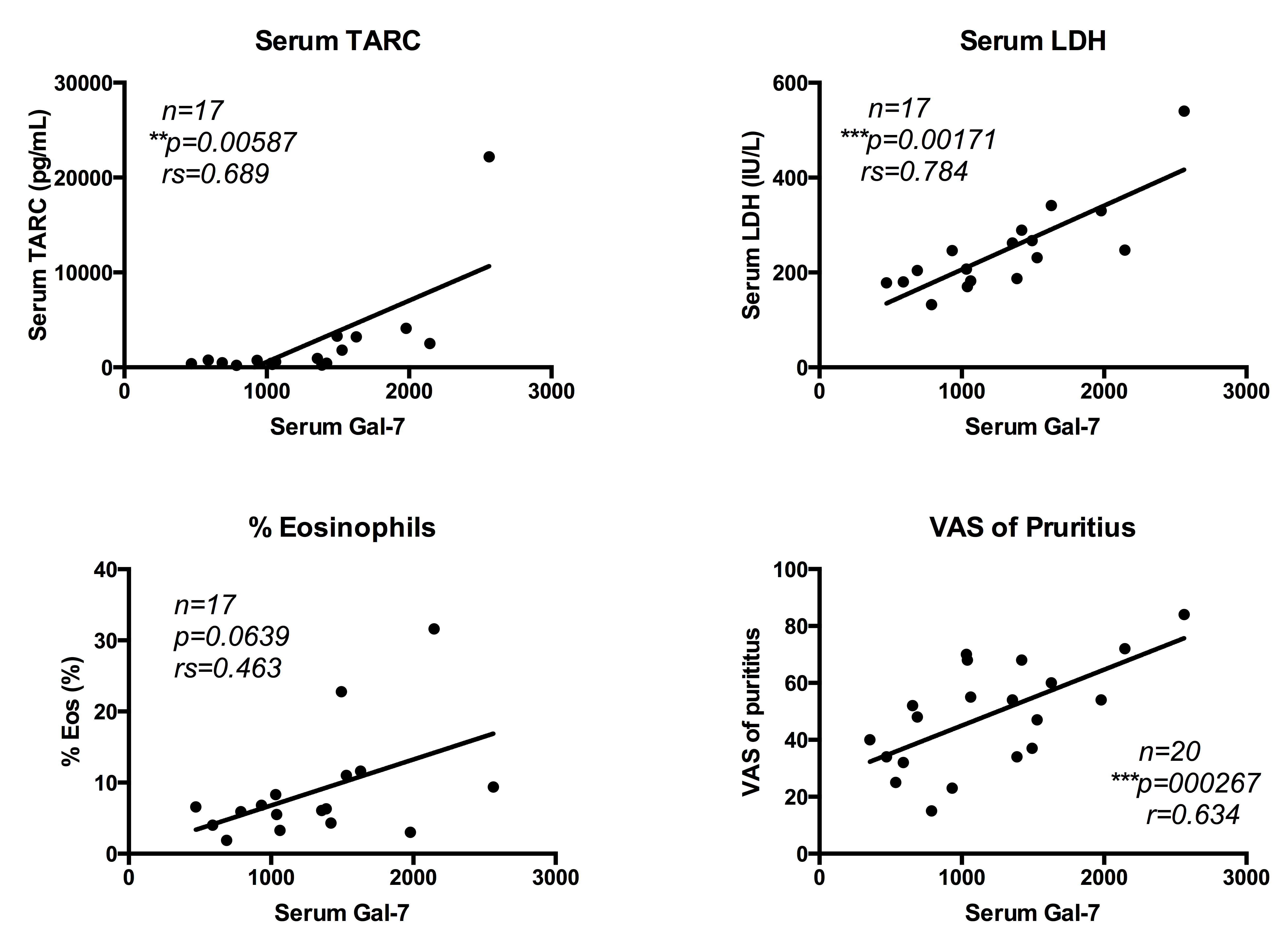

Supplement: Supplementary file 1 — Fig S1‐S2 [file CEA-50-922-s001.docx]
